# Characterization of the *Kluyveromyces marxianus* strain DMB1 *YGL157w* gene product as a broad specificity NADPH-dependent aldehyde reductase

**DOI:** 10.1186/s13568-015-0104-9

**Published:** 2015-03-03

**Authors:** Hironaga Akita, Masahiro Watanabe, Toshihiro Suzuki, Nobutaka Nakashima, Tamotsu Hoshino

**Affiliations:** Biomass Refinery Research Center, National Institute of Advanced Industrial Sciences and Technology (AIST), 3-11-32 Kagamiyama, Higashi-Hiroshima, Hiroshima 739-0046 Japan; Bioproduction Research Institute, National Institute of Advanced Industrial Sciences and Technology (AIST), 2-17-2-1 Tsukisamu-Higashi, Toyohira-ku, Sapporo, 062-8517 Japan; Department of Biological Information, Graduate School of Bioscience and Biotechnology, Tokyo Institute of Technology, 2-12-1-M6-5 Ookayama, Meguro-ku, Tokyo 152-8550 Japan

**Keywords:** Aldehyde inhibitor, BICES, GRE2, *Kluyveromyces marxianus*, Lignocellulosic biomass, Reductase

## Abstract

The open reading frame *YGL157w* in the genome of the yeast *Kluyveromyces marxianus* strain DMB1 encodes a putative uncharacterized oxidoreductase. However, this protein shows 46% identity with the *Saccharomyces cerevisiae* S288c NADPH-dependent methylglyoxal reductase, which exhibits broad substrate specificity for aldehydes. In the present study, the YGL157w gene product (KmGRE2) was purified to homogeneity from overexpressing *Escherichia coli* cells and found to be a monomer. The enzyme was strictly specific for NADPH and was active with a wide variety of substrates, including aliphatic (branched-chain and linear) and aromatic aldehydes. The optimal pH for methylglyoxal reduction was 5.5. With methylglyoxal as a substrate, the optimal temperature for enzyme activity at pH 5.5 was 45°C. The enzyme retained more than 70% of its activity after incubation for 30 min at temperatures below 35°C or at pHs between 5.5 and 9.0. In addition, the KmGRE2-overexpressing *E. coli* showed improved growth when cultivated in cedar hydrolysate, as compared to cells not expressing the enzyme. Taken together, these results indicate that KmGRE2 is potentially useful as an inhibit decomposer in *E. coli* cells.

## Introduction

The NADPH-dependent methylglyoxal reductase (EC 1.1.1.283) in *Saccharomyces cerevisiae* is termed GRE2. Using NADPH as a coenzyme, GRE2 catalyzes the stereoselective reduction of a broad range of substrates, including aldehydes and diketones, as well as aliphatic and aromatic ketones (Chen et al. 2003; Murata et al. 1985). In *S. cerevisiae*, this enzyme functions within the high osmolarity glycerol pathway (Garay-Arroyo and Covarrubias 1999), and its expression is induced by environmental conditions, including ionic, osmotic, oxidative, heat shock and heavy metal-related stresses (Garay-Arroyo and Covarrubias 1999; Krantz et al. 2004; Liu et al. 2008; Rep et al. 2001; Rutherford and Bird 2004). GRE2 also shows isovaleraldehyde reductase activity and so acts as a suppressor of filamentation (Chen et al. 2003; Hauser et al. 2007). To date, GRE2 and homologues have been purified to homogeneity from *S. cerevisiae* (Chen et al. 2003; Murata et al. 1985), *Aspergillus niger* (Inoue et al. 1988) and goat liver (Ray and Ray 1984), and their enzymatic properties have been characterized. In addition, the three-dimensional structures of the *S. cerevisiae* S288c GRE2 apo enzyme and the enzyme-NADP^+^ complex expressed in *Escherichia coli* have been solved (Guo et al. 2014). Based on its structural features, GRE2 is classified as a member of the extended short-chain-dehydrogenase/reductase superfamily (Müller et al. 2010).

*S. cerevisiae* GRE2 is currently being used as a versatile biocatalyst for the stereoselective synthesis of hydroxy compounds, which serve as building blocks in the production of pharmaceuticals and other fine chemicals (Choi et al. 2010; Ema et al. 2008; Müller et al. 2010; Park et al. 2010). Another advantageous feature of GRE2 is a decomposer in bacteria. For example, GRE2 is used for glycolaldehyde degradation during bioethanol production in *S. cerevisiae* (Jayakody et al. 2013). In addition, a *S. cerevisiae* strain overexpressing a GRE2 with site-directed mutagenesis exhibited enhanced furfural and 5-hydroxymethylfurfural (HMF) detoxification (Moon and Liu 2012). Conversely, in a *S. cerevisiae* GRE2 knockout strain growth was suppressed by environmental stress (Warringer and Blomberg 2006), and filament formation was increased in the presence of isoamyl alcohol (Hauser et al. 2007). Hence, GRE2 is regarded as a key enzyme necessary for inhibitor and stress tolerance in *S. cerevisiae*.

**Figure 1:**
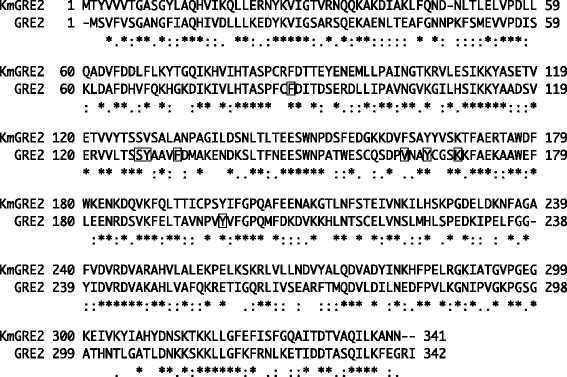
**Multiple sequence alignment of KmGRE2 and GRE2.** The sequence alignment was prepared using ClustalW (Thompson et al. 1994). Residues involved in the substrate-binding site in GRE2 are boxed.

We recently isolated *Kluyveromyces marxianus* strain DMB1, a thermotolerant yeast, from sugarcane bagasse hydrolysate and determined its genomic sequence (Suzuki et al. 2014). Within the sequence, we identified open reading frame *YGL157w*, which shows 46% identity with *S. cerevisiae* S288c GRE2 (Figure 1). In the hope of identifying a more stable GRE2 homologue, in the present study, we purified and characterized the enzyme from *K. marxianus* strain DMB1 after its overexpression in *E. coli* cells. In addition, we examined its ability to improve growth of cells cultured in cedar hydrolysate.

## Materials and Methods

### Construction of expression vectors

The plasmid pET-16b/YGL157w was constructed for production of *K. marxianus* YGL157w protein with a N-terminal hexahistidine tag. After preparation of genomic DNA from *K. marxianus* strain DMB1 (strain number: HUT7412), the *YGL157w* gene (accession number: LC016711) was amplified using PCR with KOD -plus- DNA polymerase (Toyobo, Osaka, Japan) and the primers 5′-**CAT*ATG***ACGTACGTTGTGGTTACTGGTGC-3′ (the *Nde*I site is in bold and the initiation codon is in italics) and 5′-**GGATCC***TTA*GTTGTTAGCCTTTAGTATTTG-3′ (the *Bam*HI site is in bold and termination codon is in italics). The PCR product was cloned into pTA2 (Toyobo, Osaka, Japan) and sequenced to check for PCR errors. The *YGL157w* gene was then excised from the resulting plasmid using *Nde*I and *Bam*HI and subcloned into pET-16b (Novagen, Hessen, Germany) to give pET-16b/YGL157w.

### Expression of proteins

YGL157w protein was expressed in *E. coli* BL21 (DE3) cells harboring pET-16b/YGL157w and then purified to homogeneity. The cells were grown at 37°C for 3 h in Luria-Bertani (LB) medium (1 L) containing 100 mg/L ampicillin. After inducing expression by addition of isopropyl β-D-1-thiogalactopyranoside (IPTG) to a final concentration of 1.0 mM, the culture was incubated for an additional 3 h. The cells were then harvested, suspended in 20 mM Tris–HCl buffer (pH 7.9) containing 500 mM NaCl (buffer A) and 5 mM imidazole, and disrupted by ultrasonication. The resultant lysate was clarified by centrifugation (27,500 × *g* for 15 min at 4°C), after which the supernatant was applied to a Chelating Sepharose Fast Flow column (20 mL; GE Healthcare, Buckinghamshire, UK) charged with Ni^2+^ and equilibrated with buffer A containing 5 mM imidazole. After washing the column with buffer A containing 5 mM imidazole (40 mL) and then 60 mM imidazole (60 mL), the recombinant YGL157w protein was eluted with buffer A containing 500 mM imidazole. The active fractions were pooled, concentrated using a Vivaspin 20 concentrator (10,000 MWCO, Sartorius AG, Goettingen, Germany) and loaded onto a HiLoad 26/60 Superdex 200 pg column (GE Healthcare) equilibrated with 20 mM Tris–HCl buffer (pH 8.0) containing 50 mM NaCl. The active fractions were pooled and dialyzed against 20 mM Tris–HCl buffer (pH 7.2). Finally, the dialysate was concentrated and the resultant solution was used for biochemical experiments.

Protein concentrations were determined using the Bradford method with bovine serum albumin (BSA) serving as the standard (Bradford 1976).

### Molecular mass determination

SDS-PAGE was carried out on a 10% polyacrylamide gel using the method of Laemmli (1970). EzStandard PrestainBlue (ATTO, Tokyo, Japan) was used as the molecular mass standards. The protein sample was boiled for 5 min in EzApply (ATTO). Protein bands were visualized by staining with EzStainAqua (ATTO).

The molecular mass of the native enzyme was determined by gel filtration column chromatography using a Superdex 200 Increase 10/300 GL column. Conalbumin (75 kDa), ovalbumin (43 kDa), carbonic anhydrase (29 kDa), ribonuclease A (13.7 kDa) and aprotinin (6.5 kDa) served as molecular standards (GE Healthcare).

### Assay of enzyme activity

KmGRE2 activity was measured by monitoring the decreases in the absorbance at 340 nm caused by the reduction of aldehyde, or the increases in the absorbance caused by the oxidation of alcohol. The mixture (1 mL) used for the reductive reaction contained 100 mM acetate buffer (pH 5.5), 5 mM aldehyde, 0.2 mM NADPH and YGL157w protein. The mixture (1 mL) used for the oxidative reaction contained 100 mM bicarbonate-NaOH (pH 10.0), 5 mM alcohol, 1.25 mM NADP^+^ and YGL157w protein. The reaction was started by the addition of coenzymes, and the absorbance at 340 nm was monitored at 25°C using a Shimadzu UV-2450 (Kyoto, Japan). The extinction coefficient of NADPH was 6.22 mM^−1^ cm^−1^. One unit of enzyme was defined as the amount of enzyme producing 1 μmol of NADPH per min at 25°C in the reductive reaction of methylglyoxal.

### Effects of pH and temperature on enzyme activity

The pH dependence of the reduction catalyzed by YGL157w protein was determined at 25°C using 100 mM concentrations of acetate (pH 4.0–5.5) and citrate (pH 5.5–6.5). The temperature dependence was evaluated by measuring the reductive reaction at temperatures ranging from 25 to 45°C.

### Effects of pH and temperature on enzyme stability

The effect of pH on enzyme stability was evaluated by incubating 100 nM YGL157w protein for 30 min at 35°C with 50 mM concentrations of acetate (pH 5.0–5.5), citrate (pH 5.5–6.5), phosphate (pH 6.5–8.0), borate-NaOH (pH 8.0–9.0) and bicarbonate-NaOH (pH 9.0–11.0). The enzyme solution was then rapidly cooled on ice, and the remaining activity was determined using the standard reduction assay. The thermal stability was determined by incubating YGL157w protein in 20 mM Tris–HCl buffer (pH 7.2) for 30 min at temperatures ranging from 25–45°C. The enzyme solution was then rapidly cooled on ice, and the remaining activity was determined using the standard reduction assay.

### Determination of kinetic parameters

The initial velocity of the reductive reaction was analyzed using the standard assay conditions. To determine the kinetic constants for methylglyoxal and NADPH, several concentrations of methylglyoxal (0.05–15 mM) or NADPH (0.01–0.15 mM) were used. The initial velocity was then plotted against the substrate concentration, and the *K*_m_ and *k*_cat_ values were determined by curve fitting using Igor Pro ver. 3.14 (WaveMetrics, Tigard, OR, USA).

### Preparation of hydrolysate

Lignocellulosic biomass material (Japanese cedar) was milled using a cutter mill (MKCM-3; Masuko Sangyo, Saitama, Japan), after which the resulting particles were used as the initial raw material. According to Lee et al. (2010), mechanochemical and hydrothermal pretreatment was carried out. The resulting sample was hydrolyzed using 20 FPU/g of Acremonium cellulase (Meiji Seika Pharma, Nagoya, Japan) and 40 μL/g of Optimash BG (Genencor International, Rochester, NY, USA) in 50 mM citrate buffer (pH 5.0) at 50°C and 150 rpm. After incubation for 48 h, the reaction mixture was harvested by centrifugation, and the supernatant was filtered through a 0.2 μm filter (Merck Millipore, Billerica, MA, USA). The pH of the mixture was then adjusted to 6.5, the mixture was diluted, and the resulting solution was used as the hydrolysate. Further details of the procedure are provided elsewhere (Akita et al. 2015).

### Effect of KmGRE2 expression on cell growth

The effect of KmGRE2 expression was evaluated by cultivation in a test tube using 3 mL of hydrolysate containing 0.5 mM IPTG, which was incubated at 37°C and 180 rpm. *E. coli* BL21 (DE3) cells harboring pET-16b/YGL157w or pET-16b were pregrown overnight and then diluted 1:100 with fresh hydrolysate. Cultures were monitored for cell growth at OD_600_ using an Eppendorf BioSpectrometer (Eppendorf, Hamburg, Germany).

### Quantification of sugars and aldehydes

After clarifying the culture by centrifugation and filtration, the supernatant was subjected to high performance liquid chromatography (HPLC). Quantification was performed using an Aminex HPX-87H cationic exchange column connected to an Aminex 85H Micro-Guard Column (Bio-Rad Labs, Richmond, CA, USA). The chromatographic conditions for sugars and aldehydes were as follows: mobile phase, 4.5 mM H_2_SO_4_ or 8 mM H_2_SO_4_; flow rate, 0.6 mL · min^−1^; and the column oven temperature, 65°C or 35°C. Sugars and aldehydes were detected using a Jasco RI-2031 Plus Intelligent Refractive Index Detector (Jasco, Tokyo, Japan) or a Jasco UV-2070 Plus Intelligent UV/VIS Detector at 278 nm (Jasco).

## Results

### Purification and molecular mass determination of KmGRE2

**Figure 2:**
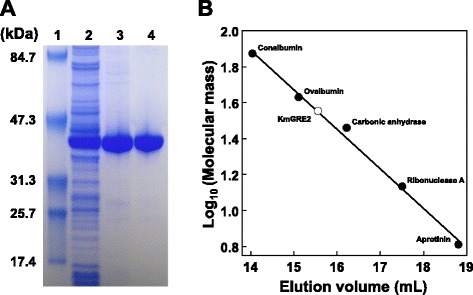
**Purification of YGL157w protein. (A)** Purification steps followed by SDS-PAGE. Proteins were separated by SDS-PAGE and visualized by EzStainAqua staining: lane 1, protein molecular size markers; lane 2, crude extract; lane 3, Chelating Sepharose Fast Flow column chromatography pool; lane 4, HiLoad 26/60 Superdex 200 pg column pool. **(B)** Determination of molecular mass using gel filtration chromatography.

After expression in 4.86 g (wet weight) of *E. coli* cells harboring pET-16b/YGL157w, YGL157w protein was purified using two successive purification steps: Chelating Sepharose Fast Flow column chromatography and gel filtration chromatography (Figure 2A). Ultimately, a pure protein was obtained with an overall yield of 82.4% (Table 1).

**Table 1:** **Purification of KmGRE2 from** ***E. coli*** **BL21 (DE3)**

**Purification step**	**Total protein (mg)**	**Total activity (U)**	**Specific activity (U/mg)**	**Yield (%)**
Crude extract	215	410	1.91	100
Chelating sepharose fast flow column	79.1	371	4.70	90.5
HiLoad 26/60 superdex 200 pg column	40.0	338	8.45	82.4

The apparent molecular mass of the YGL157w protein was determined to be about 36 kDa using Superdex 200 Increase 10/300 GL column gel filtration chromatography (Figure 2B). SDS-PAGE of the enzyme showed one major band of 40 kDa (Figure 2A), suggesting the native protein exists as a monomer.

### Substrate specificity and kinetic properties of KmGRE2

When assessed the enzymatic activity of recombinant YGL157w protein, we found that it catalyzed the reduction of linear, branched-chain and aromatic aldehydes using NADPH as the coenzyme (Table 2). High levels of activity were observed with isovaleraldehyde (C5), methylglyoxal (C3) and valeraldehyde (C5), while the lower levels were observed with octanal (C8), benzaldehyde (C7) and HMF (C6). YGL157w protein showed no activity toward *p*-anisaldehyde (C8), *p-*hydroxy benzaldehyde (C7), D-alanine, l-alanine, D-lactate, L-lactate and pyruvate. Only NADPH was utilized as the cofactor for reduction of methylglyoxal by the enzyme; NADH was not inactive. In addition, using NADP^+^ as the coenzyme, no activity was observed for oxidative reactions toward alcohol-analogs such as isoamyl alcohol, isobutanol, 2-propanol, 1-hexanol, 1-heptanol and 1-octanol under the described conditions. These results demonstrate that *YGL157w* gene encodes a NADPH-dependent GRE2, which we are calling KmGRE2.

**Table 2:** **Substrate specificity**

**Substrate**	**Relative activity (%)** ^**a**^
Isovaleraldehyde	244 ± 1.9
Methylglyoxal	100
Valeraldehyde	95.6 ± 1.6
Hexanal	81.4 ± 1.4
Heptanal	80.8 ± 2.5
Furfural	60.1 ± 1.7
Propionaldehyde	49.3 ± 0.7
Octanal	22.3 ± 1.5
Benzaldehyde	14.3 ± 2.0
HMF	1.0≧
Cinnamaldehyde	N/A^b^
Vanillin	N/A^b^

^a^Reductive activities were measured in 100 mM acetate buffer (pH 5.5) containing 3 mM substrate, 0.1 mM NADPH and 100 nM enzyme.

^b^N/A means no measurable activity. Due to the high absorbance of this substrate at 340 nm, activity was not determined under the assay conditions.

After measuring the initial rates at various methylglyoxal or NADPH concentrations, regression analyses were used to fit the data to the Michaelis-Menten equation (data not shown). The *K*_m_ and *k*_cat_ values for methylglyoxal were calculated as 0.30 ± 0.018 mM and 1.3 × 10^3^ ± 15 min^−1^, respectively. The kinetic parameters for NADPH were 0.028 ± 0.0012 mM and 1.4 × 10^3^ ± 22 min^−1^ mM^−1^, respectively. In addition, the *k*_cat_/*K*_m_ for methylglyoxal and NADPH were 4.4 × 10^3^ and 5.1 × 10^4^ min^−1^ mM^−1^, respectively. These results are similar to those of *S. cerevisiae* (Murata et al. 1985).

### Effects of pH and temperature on enzyme activity and stability

**Figure 3:**
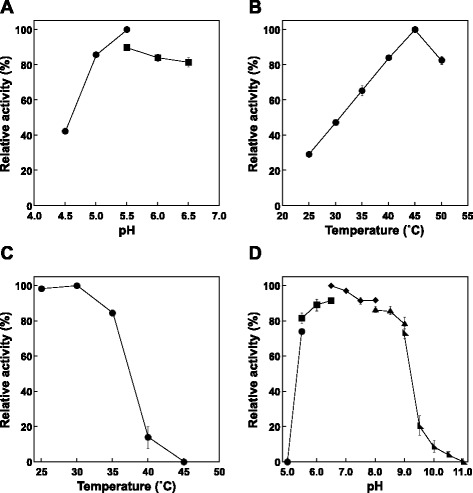
**Effects of pH and temperature on KmGRE2 activity and stability.** The markers of buffer were indicated following: circles, acetate; squares, citrate; diamonds, phosphate, isosceles triangles, borate-NaOH and right triangles, bicarbonate-NaOH. **(A)** Effect of pH on KmGRE2 activity. **(B)** Effect of temperature on KmGRE2 activity. **(C)** KmGRE2 activity after incubation for 30 min at various temperatures in the 20 mM Tris–HCl buffer (pH 7.2). **(D)** KmGRE2 activity after incubation for 30 min at 35°C in buffer solutions of various pHs. Error bars indicate SE (*n* = 3).

The effect of pH on the reduction of methylglyoxal was determined by assessing the enzyme activity at several pHs. At a temperature of 25°C, the optimum pH was about 5.5 (Figure 3A). When the temperature dependence at pH 5.5 was examined, maximum activity was observed at around 45°C (Figure 3B). Moreover, when KmGRE2 was incubated for 30 min at various temperatures in 20 mM Tris–HCl buffer (pH 7.2), KmGRE2 retained more than 80% of its activity at temperatures below 35°C (Figure 3C). On the other hand, there was a complete loss of activity when the enzyme was incubated at temperatures above 45°C. When the effect of pH on the stability of the enzyme was evaluated based on the activity remaining after incubation at 35°C for 30 min, we found that KmGRE2 retained more than 70% of its activity at pHs between 5.5 and 9.0 (Figure 3D). Somewhat disappointingly, however, KmGRE2 showed nearly the same temperature and pH stability as *S. cerevisiae* GRE2 (Park et al. 2010).

### Cell growth in hydrolysate from cedar

**Figure 4:**
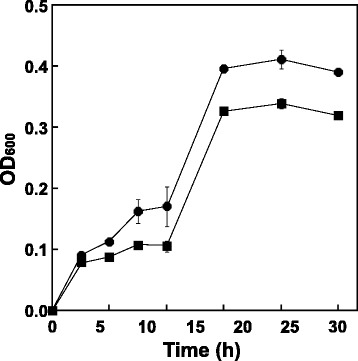
**Growth of KmGRE2-overexpressing** ***E. coli*** **cells (circles) and cells not expressing KmGRE2 (squares).** Error bars indicate SE (*n* = 3).

To assess the ability of KmGRE2 as decomposer, the effect of KmGRE2 expression on the growth of cell in cedar hydrolysate was determined by monitoring the *E. coli* growth. When cedar hydrolysate was prepared, glucose and xylose were mainly included as the sugars, whereas aldehyde inhibitors such as furfural and HMF were mainly generated. Thus, these sugars and aldehydes were detected by HPLC. The pH of hydrolysate was decided based on the optimal pH for KmGRE2 activity and the possible growth pH of *E. coli*. When cultivated in the hydrolysate under these conditions, KmGRE2-overexpressing *E. coli* showed more rapid growth than *E. coli* not expressing the enzyme (Figure 4), with enhanced furfural degradation (Table 3).

**Table 3:** **Sugar and aldehyde components in cedar hydrolysate**

**Incubation time**	**KmGRE2 expression**	**Glucose**	**Xylose**	**Furfural**	**HMF**
**(h)**		**(mM)**	**(mM)**	**(mM)**	**(mM)**
0	Not overexpressing	278.8	197.8	34.6	21.1
30	Overexpressing	262.5 ± 4.4	183.4 ± 3.4	20.5 ± 2.0	16.8 ± 0.6
	Not overexpressing	264.3 ± 1.9	184.4 ± 4.7	23.1 ± 0.2	15.9 ± 0.2

## Discussion

In the present study, we succeeded in expressing the *YGL157w* gene from *K. marxianus* strain DMB1 in *E. coli* cells and purifying the product. Characterization of the purified enzyme showed that KmGRE2 harbored strong NADPH-dependent reductive activities toward at least 10 aldehyde substrates (Tables 2). The higher activities were observed on C3 branched-chain and C3 to C7 linear aldehydes, whereas lower or no activities were detected for C8 linear aldehyde and C6 to C8 aromatic aldehydes. Conversely, *S. cerevisiae* GRE2 showed the highest activity for phenyglyoxal (C8) in the presence of NADPH (Murata et al. 1985). When we compared the amino acid sequences of KmGRE2 and *S. cerevisiae* S288c GRE2, we found that Ser127, Tyr165 and Lys169 in GRE2 were completely conserved in KmGRE2 as Ser127, Tyr165 and Lys169 (Figure 1). The three aforementioned residues in GRE2 are considered the crucial roles for the substrate dehydrogenation: Ser127 stabilizes the substrate, Tyr165 acts on a catalytic base and Lys169 facilitates the catalysis at neutral pH (Guo et al. 2014). However, two residues responsible for the substrate binding differ between the two enzymes: Phe85 and Tyr128 in GRE2 are respectively replaced by Cys85 and Val128 in KmGRE2 (Guo et al. 2014) (Figure 1). These substitutions may reduce the hydrophobic interactions for aromatic aldehydes in KmGRE2, which suggests that the molecular mechanism for substrate recognition differs between KmGRE2 and GRE2. To assess the molecular mechanism, we are now trying to obtain crystals of cofactor and/or substrate-bound KmGRE2.

The utilization of biofuel from lignocellulosic biomass holds promise as a means of abating global warming. This has prompted the development of a number of bioconversion methods for biofuel production (Akita et al. 2015; Lan and Liao 2013; Nakashima et al. 2014; da Silva et al. 2014). But while those methods produced several kinds of biofuels from hydrolysate derived of lignocellulosic biomass, the productivities and yields were often low (Akita et al. 2015; Lan and Liao 2013; Nakashima et al. 2014; da Silva et al. 2014). One of the mentioned causes of the low productivity is microbial growth inhibition by aldehyde inhibitors (Mills et al. 2009). Because aldehyde inhibitors such as furfural, HMF, glycolaldehyde, methylglyoxal and vanillin are generated mainly during the biomass hybridization process (Jarboe and Chi 2013; Jayakody et al. 2011), they are able to inhibit microbial growth and interfere with subsequent fermentation (Jayakody et al. 2011; Liu et al. 2008; Mills et al. 2009; Moon and Liu 2012). Consequently, we proposed that KmGRE2 utilizes as inhibitor decomposer. To confirm the ability of KmGRE2 to play decomposer, we assessed the effect of KmGRE2 expression on cell growth in cedar hydrolysate, production of which led to the formation of both furfural and HMF. As anticipated, the KmGRE2-overexpressing *E. coli* showed substantial growth improvement (Figure 4). We think that the growth improvement was achieved by enhanced furfural degradation, which provided for the preferable culture conditions at early culture phase. In fact, the OD_600_ of KmGRE2-overexpressing *E. coli* at 6 to 12 h were 1.3–1.6-fold higher than these of not expressing *E. coli*. On the other hand, the less activity toward HMF in KmGRE2-overexpressing *E. coli* remained unclear. The omics analysis on the metabolic response of KmGRE2-overexpressing *E. coli* may reveal this phenomenon. Recently, we developed a simple and efficient method involving biomass-inducible chromosome-based expression system (BICES) for expressing foreign genes without the use of plasmids or expensive inducers (Akita et al. 2015; Nakashima et al. 2014). This method can also be used to produce biofuels, but the productivity and yield were markedly diminished when hydrolysate from Japanese cedar as the carbon source for isobutanol production (Akita et al. 2015). We are now planning to integrate KmGRE2 gene into the genome of the *E. coli* strain involving BICES. We anticipate that this will improve growth rates, thereby increasing the productivity and yield.
